# Music and Its Inductive Power: A Psychobiological and Evolutionary Approach to Musical Emotions

**DOI:** 10.3389/fpsyg.2017.00494

**Published:** 2017-04-04

**Authors:** Mark Reybrouck, Tuomas Eerola

**Affiliations:** ^1^Faculty of Arts, Musicology Research Group, KU Leuven – University of LeuvenLeuven, Belgium; ^2^Department of Music, Durham UniversityDurham, UK

**Keywords:** induction, emotions, music and evolution, psychobiology, affective semantics, musical sense-making, adaptation

## Abstract

The aim of this contribution is to broaden the concept of musical meaning from an abstract and emotionally neutral cognitive representation to an emotion-integrating description that is related to the evolutionary approach to music. Starting from the dispositional machinery for dealing with music as a temporal and sounding phenomenon, musical emotions are considered as adaptive responses to be aroused in human beings as the product of neural structures that are specialized for their processing. A theoretical and empirical background is provided in order to bring together the findings of music and emotion studies and the evolutionary approach to musical meaning. The theoretical grounding elaborates on the transition from referential to affective semantics, the distinction between expression and induction of emotions, and the tension between discrete-digital and analog-continuous processing of the sounds. The empirical background provides evidence from several findings such as infant-directed speech, referential emotive vocalizations and separation calls in lower mammals, the distinction between the acoustic and vehicle mode of sound perception, and the bodily and physiological reactions to the sounds. It is argued, finally, that early affective processing reflects the way emotions make our bodies feel, which in turn reflects on the emotions expressed and decoded. As such there is a dynamic tension between nature and nurture, which is reflected in the nature-nurture-nature cycle of musical sense-making.

## Introduction

Music is a powerful tool for emotion induction and mood modulation by triggering ancient evolutionary systems in the human body. The study of the emotional domain, however, is complicated, especially with regard to music (Trainor and Schmidt, 2003; Juslin and Laukka, 2004; Scherer, 2004; Juslin and Västfjäll, 2008; Juslin and Sloboda, 2010; Coutinho and Cangelosi, 2011), due mainly to a lack of descriptive vocabulary and an encompassing theoretical framework. According to Sander, emotion can be defined as “an event-focused, two-step, fast process consisting of (1) relevance-based emotion elicitation mechanisms that (2) shape a multiple emotional response (i.e., action tendency, autonomic reaction, expression, and feeling” (Sander, 2013, p. 23). More in general, there is some consensus that emotion should be viewed as a compound of action tendency, bodily responses, and emotional experience with cognition being considered as part of the experience component (Scherer, 1993). Emotion, in this view, is a multicomponent entity consisting of subjective experience or feeling, neurophysiological response patterns in the central and autonomous nervous system, and motor expression in face, voice and gestures (see Johnstone and Scherer, 2000 for an overview). These components—often referred to as the *emotional reaction triad*—embrace the evaluation or appraisal of an antecedent event and the action tendencies generated by the emotion. As such, emotion can be considered as a phylogenetically evolved, adaptive mechanism that facilitates the attempt of an organism to cope with important events that affect its well-being (Scherer, 1993). In this view, changes in one of the components are integrated in order to mobilize all resources of an organism and all the systems are coupled to maximize the chances to cope with a challenging environment.

Emotions—and music-induced emotions in particular, —are thus difficult to study adequately and this holds true also for the idiosyncrasies of individual sense-making in music listening. Four major areas, however, have significantly advanced the field: (i) the development of new research methods (continuous, real-time and direct recording of physiological correlates of emotions), (ii) advanced techniques and methods of neuroscience (including fMRI, PET, EEG, EMG and TMS), (iii) theoretical advances such as the distinction between felt and perceived emotions and acknowledgment of various induction mechanisms, and (iv) the adoption of evolutionary accounts. The development of new research methods, in particular, has changed dramatically the field, with seminal contributions from neuropsychology, neurobiology, psychobiology and affective neuroscience. There is, however, still need of a conceptual and theoretical framework that brings all findings together in a coherent way.

**FIGURE 1:**
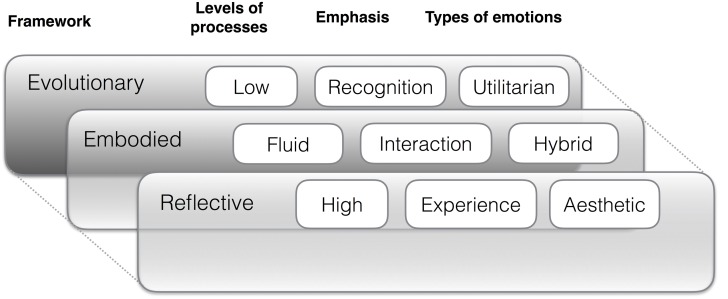
**Conceptual framework of emotion processes involved in music listening**.

In order to address this issue, we organize our review of the field on three broad theoretical frameworks that are indispensable for the topic, namely an evolutionary, embodied and reflective one (see **Figure 1**). Within these frameworks, we focus on the levels and emphasis of the processes involved and connect the types of emotion conceptualizations involved to these frameworks. For instance, the levels of processes are typically divided into low-level and high-level processes, the emphasis of the emotion ranges from recognition to experience of emotion, and the types of emotions involved in these frameworks are usually tightly linked to the levels and emphases. Emotion recognition, e.g., is typically associated with utilitarian emotions, whereas higher level and cognitively mediated reflective emotions that are largely the product of emotion experience might be better conceptualized by aesthetic emotions. The embodied framework does break these dichotomies of high and low and recognition and experience in postulating processes that are flexible, fluid and driven through modality-specific systems that emphasize the interaction between the events offered by the environment, the sensory processes and the acquired competencies for reacting to them in an appropriate fashion.

In what follows, we will start from an evolutionary approach to musical emotions—defining them to some extent as adaptations—, looking thereafter toward the contributions from affective semantics and the embodied framework for explaining musical emotions from a neuroscientific perspective. We then move onto some psychobiological claims to end with addressing the issue of modulation of emotions by aesthetic experience. In doing so we will look at some conceptual challenges associated with emotions before moving onto emotional meanings in music with the aim to connect experience and meaning-making in the context of emotions to the functions of emotions within an evolutionary perspective. The latter, finally, will be challenged to some extent.

## Evolutionary Claims: Emotions as Adaptations

The neurosciences of music have received a lot of attention in recent research. The *neuroaesthetics* of music, however, remains still somewhat undeveloped as most of the experiments that have been conducted aimed at studying the neural effects on perceptual and cognitive skills rather than on aesthetic or affective judgments (Brattico and Pearce, 2013). Psychology and neuroscience, up to now, have been preoccupied mostly with the *cortico-cognitive systems* of the human brains rather than with *subcortical-affective* ones. Affective consciousness, as a matter of fact, needs to be distinguished from more cognitive forms which generate propositional thoughts about the world. These evolutionary younger cognitive functions add an enormous richness to human emotional life but they neglect the fact that the “energetic” engines for affect are concentrated sub-neocortically. Without these ancestral emotional systems of our brains, music would probably become a less meaningful and desired experience (Panksepp and Bernatzky, 2002; Panksepp, 2005).

In order to motivate these claims, there is need of bottom–up evolutionary, and mainly adaptationist proposals in search of the origins of aesthetic experiences of music, starting from the identification of universal musical features that are observable in all cultures of the world (Brattico et al., 2009–2010). The exquisite sensitivity of our species to emotional sounds, e.g., may function as an example of the survival advantage conferred to operate within small groups and social situations where reading another person’s emotional state is of vital importance. This is akin to privileged processing of human faces, which is another highly significant social signal that has been a candidate for evolutionary selection. Processing affective sounds, further, is assumed to be a crucial element for the affective-emotional appreciation of music, which, in this view, can arouse basic emotional circuits at low hierarchical levels of auditory input (Panksepp and Bernatzky, 2002).

Music has been considered from an *evolutionary* perspective in several lines of research, ranging from theoretical discussions (see Brattico et al., 2009–2010; Cross, 2009–2010; Lehman et al., 2009–2010; Livingstone and Thompson, 2009–2010; Honing et al., 2015), to biological (Peretz et al., 2015) and cross-cultural (Trehub et al., 2015), and cross-species evidence (Merchant et al., 2015). Although these various accounts have not fully unpacked the functional role of emotions in the origins of music, certain agreed positions have emerged. For instance, music is conceived as a universal phenomenon with *adaptive power* (Wallin et al., 2000; Huron, 2003; Justus and Hutsler, 2005; McDermott and Hauser, 2005; Dissanayake, 2008; Cross, 2009–2010). Neuroscientists as LeDoux (1996) and Damasio (1999) have argued that emotions did not evolve as conscious feelings but as adaptive bodily responses that are controlled by the brain. LeDoux (1989, 1996), moreover, has proposed two separate neural pathways that mediate between sensory stimuli and affective responses: a *low road* and a *high road*. The “low road” is the subcortical pathway that transmits emotional stimuli directly to the amygdala—a brain structure that regulates behavioral, autonomic and endocrine responses—by way of connections to the brain stem and motor centers. It bypasses higher cortical areas which may be involved in cognition and consciousness and triggers emotional responses (particularly fear responses) without cognitive mediation. As such, it involves *reactive activity* that is pre-attentive, very fast and automatic, with the “startle response” as the most typical example (Witvliet and Vrana, 1996; Błaszczyk, 2003). Such “primitive” processing has considerable adaptive value for an organism in providing levels of elementary forms of decision making which rely on sets of neural circuits which do the deciding (Damasio, 1994; Lavender and Hommel, 2007). It embraces mainly physiological constants, such as the induction or modification of arousal as well as bodily reactions with a whole range of autonomic reactions. The “high road,” on the contrary, passes through the amygdala to the higher cortical areas. It allows for much more fine-grained processing of stimuli but operates more slowly.

Primitive processing is to be found also in the processing of emotions, which, at their most elementary level, may behave as reflexes in their operation. Occurring with rapid onset, through automatic appraisal and with involuntary changes in physiological and behavioral responses (Peretz, 2001), this level is analogous to the functioning of innate *affect programs* (Griffiths, 1997), which can be assigned to an inherited subcortical structure that can instruct and control a variety of muscles and glands to respond with unique patterns of activity that are characteristic of a given affect (Tomkins, 1963). Defined in this way, affect programs related to music should be connected to rapid, automatic responses caused by sudden loud sounds (brain stem reflex in the BRECVEMA model, see below). However, a broader interpretation of affect programs as being embodied and embedded in body states and their simulations would put the majority of the emotions into this elementary level (Niedenthal, 2007). In our view, such a broadened embodied view may be a more fruitful way of mapping out the links between the stimuli and emotions than the rather narrow definition of affect programs.

Musically induced emotions, considered at their lowest level, can be conceived partly as *reactive behavior* that points into the direction of automatic processing, involving a lot of biological regulation that engages evolutionary older and less developed structures of the brain. They may have originated as adaptive responses to acoustic input from threatening and non-threatening sounds (Balkwill and Thompson, 1999) which can be considered as quasi-universal reactions to auditory stimuli in general and by extension also to sounding music. Dealing with music, in this view, is to be subsumed under the broader category of “coping with the sounds” (Reybrouck, 2001, 2005). It means also that the notion of musicality, seen exclusively as an evolved trait that is specifically shaped by natural selection, has been questioned to some extent, in the sense that the role of learning and culture have been proposed as possible alternatives (Justus and Hutsler, 2005).

From an *evolutionary* perspective, music has often been viewed as a by-product of natural selection in other cognitive domains, such as, e.g., language, auditory scene analysis, habitat selection, emotion, and motor control (Pinker, 1997; see also Hauser and McDermott, 2003). Music, then, should be merely *exaptive*, which means that is only an evolutionary by-product of the emergence of other capacities that have direct adaptive value. As such, it should have no role in the survival as a species but should have been derived from an optimal instinctive sensitivity for certain sound patterns, which may have arisen because it proved adaptive for survival (Barrow, 1995). Music, in this view, should have exploited parasitically a capacity that was originally functional in primitive human communication [still evident in speech, note the similarity of affective cues in speech and music (Juslin and Laukka, 2003)] but that fell into disuse with the emergence of finer shades of differentiation in sound pattern that emerged with the emergence of music (Sperber, 1996). As such, processes other than direct adaptation, such as cultural *transmission* and *exaptation*, seem suited to complement the study of biological and evolutionary bases of dealing with music (Tooby and Cosmides, 1992; Justus and Hutsler, 2005, see also below).

A purely *adaptationist* point of view has thus been challenged with regard to music. In a rather narrow description, the notion of adaptation revolves around the concepts of *innate constraint* and *domain specificity*, calling forth also the *modularity approach* to cognition (Fodor, 1983, 1985), which states that some aspects of cognition are performed by mental modules or mechanisms that are specific to the processing of only one kind of information. They are largely innate, fast and unaffected by the content of other representations, and are implemented by specific localizable brain regions. Taken together, such qualities can be referred to as “domain specificity,” “innate constraints,” “information encapsulation” and “brain localization” (see Justus and Hutsler, 2005).

Several attempts have been made to apply the modular approach to the domain of music. It has been shown, e.g., that the representation of pitch in terms of a tonal system can be considered as a module with specialized regions of the cortex (Peretz and Coltheart, 2003). Much of music processing occurs also implicitly and automatically, suggesting some kind of information encapsulation. It can be questioned, however, whether the relevant cortical areas are really domain-specific for music. The concept of modularity, moreover, has been critized, as different facets of modularity are dissociable with the introduction of the concept of *distributivity* as a possible alternative (Dick et al., 2001). One way in which this dissociation works is the discovery of emergent modules in the sense that predictable regions of the cortex may become informationally encapsulated and/or domain specific, without the outcome having been planned by the genome (Karmiloff-Smith, 1992). The debate concerning the innateness of music processing, however, is not conclusive. A lot of research still has to be done to address the ways in which a domain is innately constrained (Justus and Hutsler, 2005). Most of the efforts, up to now, have concentrated on *perception* and *cognition*, with the importance of octave equivalence and other simple pitch ratios, the categorization of discrete tone categories within the octave, the role of melodic contour, tonal hierarchies and principles of grouping and meter as possible candidate constraints. Music, however, is not merely a cognitive domain but calls forth experiential claims as well, with many connections with the psychobiology and neurophysiology of affection and emotions. *Affective neuroscience* has already extended current knowledge of the emotional brain to some extent (Davidson and Sutton, 1995; Panksepp, 1998; Sander, 2013), but a lot of work still has to be done.

Dealing with musically induced emotions, further, can be approached from different scales of description: the larger evolutionary scale (*phylogeny*) and the scale of individual human development (*ontogeny*).

An abundance of empirical evidence has been gathered from developmental (newborn studies and infant-directed speech) (Trehub, 2003; Falk, 2009) and comparative research between humans and non-human animals (referential emotive vocalizations and separation calls). It has been shown, e.g., that evolution has given *emotional sound* special time-forms that arise from frequency and amplitude modulation of relatively simple acoustic patterns (Panksepp, 2009–2010). As such, there are means of sound communication in general which are partly shared among living primates and other mammals (Hauser, 1999) and which are the result of brain evolution with the appearance of separate layers that have overgrown the older functions without actually replacing them (Striedter, 2005, 2006). By using sound carriers, humans seem to be able to transmit information such as spatial location, structure of the body, sexual attractiveness, emotional states, cohesion of the group, etc. Some of it is present in all sound messages, but other kinds of information seem to be restricted to specific ways of sound expression (Karpf, 2006). The communicative accuracy of these sets of information, however, has been rarely if at all studied except for emotion states.

This is the case even more for singing, as a primitive way of music realization that was probably previous to any kind of instrumental music making (Geissmann, 2000; Mithen, 2006) and which contains different degrees of motor, emotional and cognitive elements which are universal for us as a species. Generalizing a little, there are special forms of human sound expression that allow communication with other species and reactions to sound stimuli that are similar to those of animals. On the other hand, there seems to be a set of specific sound features belonging exclusive to man—music features such as, e.g., tonality and isometry—, which are strongly connected with emotion expression but which are absent in other kinds of human sound communication (see Gorzelañczyk and Podlipniak, 2011). This is obvious in speech and music and even in some animal vocalizations. The acoustic measures of speech, e.g., can be subdivided into four categories: time-related measures (temporal sequence of different types of sound and silence as carriers of affective information), intensity-related measures (amount of energy in the speech signal), measures related to fundamental frequency (F_0_ base level and F_0_ range; relative power of fundamental frequency and the harmonics F_1_, F_2_, etc.), and more complicated time-frequency-energy measures (specific patterns of resonant frequencies such as formants). Three of them are linked to the perceptual dimensions of speech rate, loudness and pitch, the fourth is related to the perceived timbre and voice quality (Johnstone and Scherer, 2000). Taken together, these measures have made it possible to measure the encoding of vocal affect, at least for some commonly studied emotions such as stress, anger, fear, sadness, joy, disgust, and boredom with most consistency in the findings for arousal. The search for emotion-specific acoustic patterns with similar arousal, however, is still a subject of ongoing research (Banse and Scherer, 1996; Eerola et al., 2013).

## Affective Semantics and the Embodied Framework

Music can be considered as a sounding and temporal phenomenon, with the experience of time as a critical factor for musical sense-making. Such an *experiential approach* depends on perceptual bonding and continuous processing of the sound (Reybrouck, 2014, 2015). It can be questioned, in this regard, whether the standard self-report instruments of induced emotions (Eerola and Vuoskoski, 2013) are tapping onto the experiential level or whether that experiential level is inaccessible by such methods, although it may be partially accessible by introspection and verbalization. To address this question, a distinction should be made between the recognition of emotions and the emotions as felt. The former can be considered as a “cognitive-discrete” process which is reducible to categorical assessments of the affective qualia of sounds; the latter calls forth a continuous experience which entails a conception of “music-as-felt” rather than a disembodied approach to musical meaning (Nagel et al., 2007; Schubert, 2013). Though the distinction has received already some attention, there is still need of a conceptual and theoretical framework that brings together current knowledge on perceived and induced emotions in a coherent way. Ways of handling time and experience in music and emotion research up to now have not been neglected (Jones, 1976; Jones and Boltz, 1989) with a significant number of continuous rating studies (Schubert, 2001, 2004), but the study of time has not been the real strength of this research. It can be argued, therefore, that time is not merely an empty perception of duration. It should be considered, on the contrary, as one of the contributing dimensions in the study of emotions in their dynamic form. It calls forth the role of *affective semantics*—a term coined by Molino (2000)—, which aims at describing the meaning of something not in terms of abstract and emotionally neutral cognitive representations, but in a way that is dependent mainly on the integration of emotions (Brown et al., 2004; Menon and Levitin, 2005; Panksepp, 2009–2010). Musical semantics, accordingly, is in search not only of the *lexico-semantic* but also of the *experiential* dimension of meaning, which, in turn, is related to the affective one. Affective semantics, as applied to music, should be able to recognize the emotional meanings which particular sound patterns are trying to convey. It calls forth a continuous rather than a discrete processing of the sounds in order to catch the expressive qualities that vary and change in a dynamic way. Emotional expressions, in fact, are not homogeneous over time, and many of music’s most expressive qualities relate to structural changes over time, somewhat analogous to the concept of *prosodic contours* which is found in vocal expressions (Banse and Scherer, 1996; Scherer, 2003; Belin et al., 2008; Hawk et al., 2009; Sauter et al., 2010; Lima et al., 2013).

The strongest arguments for the introduction of affective semantics in music emotion research come from the developmental perspective (Trainor and Schmidt, 2003): caregivers around the world sing to infants in an *infant-directed* singing style—using both lullaby and playsong style—which is probably used in order to express emotional information and to regulate the infant’s state. This style—also known as *motherese*—is distinct from other types of singing and young infants are very responsive to it. Additional empirical grounding, moreover, comes from primate vocalizations, which are coined as *referential emotive vocalizations* (Frayer and Nicolay, 2000) and *separation calls* (Newman, 2007). Embracing a body of calls that serve a direct emotive response to some object or events in the environment, they exhibit a dual acoustic nature in having both a referential and emotive meaning (Briefer, 2012).

It is arguable, further, that the affective impact of music could be traced back to similar grounds, being generated by the modulation of sound with a close connection between primitive *emotional dynamics* and the essential *dynamics of music*, both of which appear to be biologically grounded as innate release mechanisms that generate instinctual emotional actions (Burkhardt, 2005; Panksepp, 2009–2010; Coutinho and Cangelosi, 2011). Along with the evolved appreciation of temporal progressions (Clynes and Walker, 1986) they can generate, relive, and communicate emotion intensity, helping to explain why some emotional cues are so easily rendered and recognized through music. This can be seen in the rare cases, where music expressing particular emotions have been exposed to listeners from distinct cultures, at least concerning basic or primary emotions, such as happy, sad, and angry (Balkwill and Thompson, 1999; Fritz et al., 2009). The case seems to be more complicated, however, with regard to secondary or aesthetic emotions such as, e.g., spirituality and longing (Laukka et al., 2013).

As such, there is more to music than the recognition of discrete elements and the way they are related to each other. As important is a description of “music-as-felt,” somewhat analogous to the distinction which has been made between the *vehicle* and the *acoustic mode* of sense-making (Frayer and Nicolay, 2000). The latter refers to particular sound patterns being able to convey emotional meanings by relying on the immediate, on-line emotive aspect of sound perception and production and deals with the emotive interpretation of musical sound patterns; the vehicle mode, on the other hand, involves referential meaning, somewhat analogous to the lexico-semantic dimension of language, with arbitrary sound patterns as vehicles to convey symbolic meaning. It refers to the off-line, referential form of sound perception and production, which is a representational mode of dealing with music that results from the influence of human linguistic capacity on music cognition and which reduces meaning to the perception of “disembodied elements” that are dealt with in a propositional way.

The online form of sound perception—the acoustic mode—is somewhat related to the Clynes’ concept of *sentic modulation* (Clynes, 1977), as a general modulatory system that is involved in conveying and perceiving the intensity of emotive expression by means of three graded spectra of tempo modulation, amplitude modulation, and register selection, somewhat analogous to the well-known rules of prosody. In addition, there is also timbre as a separate category (Menon et al., 2002; Eerola, 2011), which represents three major dimensions of sounds, namely the temporal (attack time), spectral (spectral energy distribution) and spectro-temporal (spectral flux) (Eerola et al., 2012, p. 49). The very idea of sentic modulation has been taken up in recent studies about emotional expression that is conveyed by *non-verbal vocal expressions*. Examples are the modifications of prosody during expressive speech and non-linguistic vocalizations such as breathing sounds, crying, hums, grunts, laughter, shrieks, and sighs (Juslin and Laukka, 2003; Scherer, 2003; Thompson and Balkwill, 2006; Bryant and Barrett, 2008; Pell et al., 2009; Bryant, 2013) and non-verbal affect vocalizations (Bradley and Lang, 2000; Belin et al., 2008; Redondo et al., 2008; and Reybrouck and Podlipniak, submitted, for an overview). Starting from the observation that the body usually responds physically to an emotion, it can be claimed that physiological responses act as a trigger for appropriate actions with the motor and visceral systems acting as typical manifestations, but other modalities are possible as well. As such, the concept of sentic modulations can be related to Niedenthal’s embodied approach to multimodal processing, surpassing the muscles and the viscera in order to focus on modality-specific systems in the brain perception, action and introspection that are fast, refined and flexible. They can even be reactivated without their output being observable in overt behavior with embodiment referring both to actual bodily states and simulations of the modality-specific systems in the brain (Niedenthal et al., 2005; Niedenthal, 2007).

The musical-emotional experience, further, has received much impetus from theoretical contributions and empirical research (Eerola and Vuoskoski, 2013). Impinging upon the body and its physiological correlates, it calls forth an embodied approach to musical emotions which goes beyond the standard cognitivist approach. The latter, based on appraisal, representation and rule-based or information-processing models of cognition, offers rather limited insights of what a musical-emotional experience entails (Schiavio et al., 2016; see also Scherer, 2004 for a critical discussion). Alternative embodied/enactive models of mind—such as the “4E” model of cognition (*embodied*, *embedded*, *enactive*, and *extended*, see Menary, 2010)—have challenged this approach by emphasizing meaning-making as an ongoing process of dynamic interactivity between an organism and its environment (Barrett, 2011; Maiese, 2011; Hutto and Myin, 2013). Relying on the basic concept of “enactivism” as a cross-disciplinary perspective on human cognition that integrates insights from phenomenology and philosophy of mind, cognitive neuroscience, theoretical biology, and developmental and social psychology (Varela et al., 1991; Thompson, 2007; Stewart et al., 2010), enactive models understand cognition as embodied and perceptually guided activity that is constituted by circular interactions between an organism and its environment. Through continuous sensorimotor loops (defined by real-time perception/action cycles), the living organism—including the music listener/performer—enacts or brings forth his/her own domain of meaning (Reybrouck, 2005; Thompson, 2005; Colombetti and Thompson, 2008) without separation between the cognitive states of the organism, its physiology, and the environment in which it is embedded. Cognition and mind, in this view, originate in a continuous interplay between an organism and its environment as an evolving dynamic system (Hurley, 1998).

Starting from the observation that the body usually responds physically to an emotion, it can be claimed, further, that physiological responses act as a trigger for appropriate actions with the motor and visceral systems acting as typical manifestations. Other modalities, however, are possible as well., as exemplified in Niedenthal’s embodied approach to multimodal processing, surpassing the muscles and the viscera in order to focus on modality-specific systems in the brain—perception, action and introspection—that are fast, refined and flexible. They can even be reactivated without their output being observable in overt behavior. Embodiment, then, is referring both to actual bodily states or simulations of the modality-specific systems in the brain (Niedenthal et al., 2005; Niedenthal, 2007).

## Induction of Emotions: Psychobiological Claims

Music may be considered as something that catches us and that induces several reactions beyond conscious control. As such, it calls forth a deeper affective domain to which cognition is subservient, and which makes the brains such receptive vessels for the emotional power of music (Panksepp and Bernatzky, 2002). The auditory system, in fact, evolved phylogenetically from the *vestibular system*, which contains a substantial number of acoustically responsive fibers (Koelsch, 2014). It is sensitive to sounds and vibrations—especially those of loud sounds with low frequencies or with sudden onsets—and projects to the reticular formation and the parabrachial nucleus, which is a convergence site for vestibular, visceral and autonomic processing. As such, subcortical processing of sounds gives rise not only to auditory sensations but also to muscular and autonomic responses. It has been shown, moreover, that intense hedonic experiences of sound and pleasurable aesthetic responses to music are reflected in the listeners’ autonomic and central nervous systems, as evidenced by objective measurements with polygraph, EEG, PET or fMRI (Brattico et al., 2009–2010). Though these measures do not always differentiate between specific emotions, they indicate that the *reward syste*m can be heavily activated by music (Blood and Zatorre, 2001; Salimpoor et al., 2015). But other brain structures can be activated as well, more particularly those brain structures that are crucially involved in emotion, such as the amygdala, the nucleus accumbens, the hypothalamus, the hippocampus, the insula, the cingulate cortex and the orbitofrontal cortex (Koelsch, 2014).

Emotional reactions to music, further, activate the same cortical, subcortical and autonomic circuits, which are considered as the essential survival circuits of biological organisms in general (Blood and Zatorre, 2001; Trainor and Schmidt, 2003; Salimpoor et al., 2015). The subcortical processing affects the body through the basic mechanisms of chemical release in the blood and the spread of neural activation. The latter, especially, invites listeners to react bodily to music with a whole bunch of autonomic reactions such as changes in heart rate, respiration rate, blood flow, skin conductance, brain activation patterns, and hormone release (oxytocin, testosterone), all driven by the phylogenetically older parts of the nervous system (Ellis and Thayer, 2010). These reactions can be considered the “physiological correlates” of listening (see Levenson, 2003, for a general review), but the question remains whether such measures provide sufficient detailed information to distinguish musically induced physiological reactions from mere physiological reactions to emotional stimuli in general (Lundqvist et al., 2009). Recent physiological studies have shown that pieces of music that express different emotions may actually produce distinct physiological reactions in listeners (see Juslin and Laukka, 2004 for a critical review). It has been shown also that performers are able to communicate at least five emotions (happiness, anger, sadness, fear, tenderness) with this proviso that this communication operates in terms of broader emotional categories than the finer distinctions which are possible within these categories (Juslin and Laukka, 2003). Precision of communication, however, is not a primary criterion by which listeners value music and reliability is often compromised for the sake of other musical characteristics. Physiological measures may thus be important, but establishing clear-cut and consistent relationships between emotions and their physiological correlates remains difficult, though some studies have received some success in the case of few basic emotions (Juslin and Laukka, 2004; Lundqvist et al., 2009).

Music thus has *inductive power.* It engenders physiological responses, which are triggered by the central nervous system and which are proportional to the way the information has been received, analyzed and interpreted through instinctive, emotional pathways that are ultimately concerned with maintaining an internal environment that ensures survival (Schneck and Berger, 2010). Such dynamically equilibrated and delicately balanced internal milieu (*homeostasis*), together with the physiological processes which maintain it, relies on finely tuned control mechanisms that keep the body operating as closely as possible to predetermined baseline physiological quantities or *reference set-points* (blood pressure, pulse rate, breathing rate, body temperature, blood sugar level, pH, fluid balance, etc.). Sensory stimulation of all kinds can change and disturb this equilibrium and invite the organism to adapt these basic reference points, mostly after persisting and continuous disturbances that act as environmental or driving forces to which the organism must adapt. There are, however, also short term immediate reactions to the music as a driving force, as evidenced from neurobiological and psychobiological research that revolves around the central axiom of *psychobiological equivalence* between percepts, experience and thought (Reybrouck, 2013). This axiom addresses the central question whether there is some lawfulness in the coordination between sounding stimuli and the responses of music listeners in general. A lot of empirical support has been collected from studies of *psychophysical dimensions* of music as well as *physiological reactions* that have shown to be their correlates (Peretz, 2001, 2006; Scherer and Zentner, 2001; Menon and Levitin, 2005; van der Zwaag et al., 2011). Psychophysical dimensions, as considered in a musical context, can be defined as any property of sound that can be perceived independently of musical experience, knowledge, or enculturation, such as, e.g., speed of pulse or tempo. A distinction should be made, however, between the *psychophysics* of perception and the *psychobiology* of the bodily reactions to the sounds. The psychophysics features suggest a reliable correlation between acoustic signals and their perceptual processing, with a special emphasis on the study of how individual features of music contribute to its emotional expression, embracing psychoacoustic features such as loudness, roughness and timbre (Eerola et al., 2012). The psychobiological claims, on the other hand, are still subject of ongoing research. Some of them can be subsumed under the sensations of *peak experience*, *flow* and *shivers* or *chills* (Panksepp and Bernatzky, 2002; Grewe et al., 2007; Harrison and Loui, 2014) as evidence for particularly strong emotional experiences with music (Gabrielsson and Lindström, 2003; Gabrielsson, 2010). Such intensely pleasurable experiences are straightforward to be recorded behaviorally and have the additional advantage of producing characteristic physiological markers including changes in heart rate, respiration amplitude, and skin conductance (e.g., Blood and Zatorre, 2001; Sachs et al., 2016). They are associated mainly with changes in the autonomic nervous system and with metabolic activity in the cerebral regions, such as ventral striatum, amygdala, insula, and midbrain, usually devoted to motivation, emotion, arousal, and reward (Blood and Zatorre, 2001). Their association with subcortical structures indicates also their possible association with ancestral behavioral patterns of the prehistoric individual, making them relevant for the evaluation of the evolutionary hypothesis on the origin of aesthetic experience of music (Brattico et al., 2009–2010). Such peak experiences, however, are rather rare and should not be taken as the main starting point for a generic comparative perspective on musical emotions. Some broader *vitality effects*, such as those exemplified in the relations between personal feelings and the dynamics of infant’s movements and the sympathetic responses by their caregivers in a kind of mutual attunement (Stern, 1985, 1999; see also Malloch and Trevarthen, 2009), as well as the creation of tensions and expectancies may engender also some music-specific emotional reactions. The general assumption, then, is that musically evoked reactions emerge from “presemantic acoustic dynamics” that evolved in ancient times, but that still interact with the intrinsic emotional systems of our brains (Panksepp, 1995, p. 172)

## An Integrated Framework of Music Emotions and their Underlying Mechanisms

**FIGURE 2:**
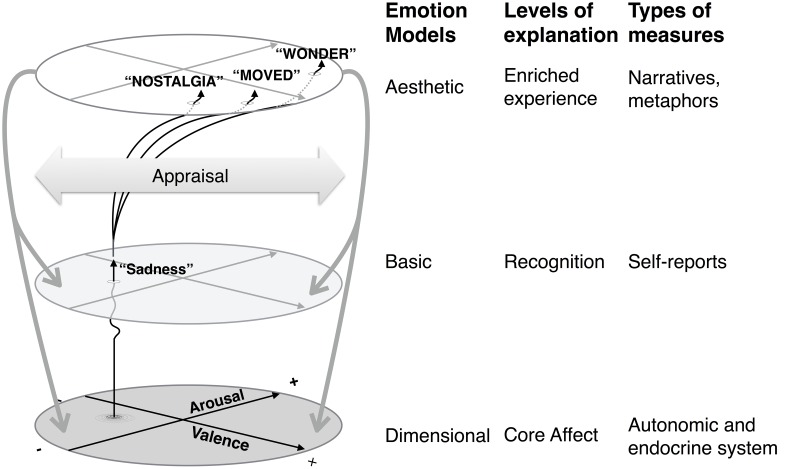
**Visualization of a hybrid model of emotions as applied to music**.

What are these presemantic acoustic dynamics? Here we should make a distinction between the *structural features* of the music which induce emotions and their *underlying mechanisms*. As to the first, musical cues such as mode, followed by tempo, register, dynamics, articulation, and timbre (Eerola et al., 2013) seem to be important, at least in Western music. Increases in perceived complexity, moreover, has been shown also to evoke arousal (Balkwill and Thompson, 1999). Being grounded in the dispositional machinery of individual music users these features may function as universal cues for the emotional evaluation of auditory stimuli in general. Much more research, however, is needed in order to trace their underlying mechanisms. A major attempt has been made already by Juslin and Västfjäll (2008) and Liljeström et al. (2013) who present a framework that embraces eight basic mechanisms (brain stem reflexes, rhythmic entrainment, evaluative conditioning, emotional contagion, visual imagery, episodic memory, musical expectancy and aesthetic judgment—commonly referred to as BRECVEMA). In addition to these mechanisms, an *integrated framework* has been proposed also by Eerola (2017), with low-level measurable properties being capable of producing highly different higher-level conceptual interpretations (see **Figure 2**). Its underlying machinery is best described in dimensional terms (core affects as valence and arousal) but conscious interpretations can be superposed on them, allowing a categorical approach that relies on higher-level conceptual categories as well. As such, the model can be considered a hybrid model that builds on these existing emotion models and attempts to clarify the levels of explanations of emotions and the typical measures related to these layers of explanations. Although this is a simplification of a complex process, the purpose is to emphasize the disparate conceptual issues brought under the focus at each different level, which is a notion put forward in the past (e.g., Leventhal and Scherer, 1987). The types of measures of emotions alluded to in the model are not merely alternative instruments but profoundly different ontological stances which capture biological reductionism (all physiological responses), psychological (all behavioral responses including self-reports) and phenomenological (various experiential including narratives and metaphors) perspectives.

The dimensional perspective on emotions has fostered already a long program of research with objectless dimensions such as pleasure–displeasure (pleasure or valence) and activation–deactivation (arousal or energy). Their combination—called *core affect*—can be considered as a first primitive that is involved in most psychological events and makes them “hot” or emotional. Involving a pre-conceptual process, a neurophysiological state, core affect is accessible to consciousness as a simple non-reflective feeling, e.g., feeling good or bad, feeling lethargic or energized. Perception of the affective quality is the second primitive. It is a “cold” process which is made hot by being combined with a change in core affect (Russell, 2003, 2009).

The dimensional approach has been challenged to some extent. Eerola’s *hybrid model* (Eerola, 2017) assigns three explanatory levels of affects, starting from low level sensed emotions (core affect), proceeding over perceived or recognized emotions (basic emotions), and ending with experienced and felt emotions (high-level complex emotions). It takes as the lowest level core affect, as a neurophysiological state which is accessible to consciousness as a simple primitive non-reflective feeling (Russell and Barrett, 1999). It reflects the idea that affects arise from the core of the body and neural representations of the body state. The next higher level organizes emotions by conceiving of them in terms of discrete categories such as fear, anger, disgust, sadness, and surprise (Matsumoto and Ekman, 2009; and Sander, 2013 for a discussion of number and label of the categories). Both levels have furthered an abundance of theoretical and empirical research with a focus on the development of emotion taxonomies which all offer distinct ways to tackle musical emotions. Both the dimensional and basic emotions model, however, seem to overlap considerably, and this holds true especially for artworks and objects in nature (Eerola and Vuoskoski, 2011) which are not always explained in terms of dimensions or discrete patterns of emotions that are involved in everyday survival (Sander, 2013). As such, there is also a level beyond core affects and the perception of basic emotions which is not reducible to mere reactions to the environment, and that encompasses complex emotions that are more contemplative, reflected and nuanced, somewhat analogous to other complex emotions such as moral, social and epistemic ones (see below).

While such a hybrid model may reconcile some of the discrepancies in the field, its main contribution is to make us aware of how the conceptual level of emotions under the focus lends itself to different mechanisms, emotion labels and useful measures. The shortcoming of the model is an impression that it offers a way to reduce complex, aesthetic emotions into simpler basic emotions and the latter into underlying core affects. Whilst some of such trajectories could be traced from the lowest to highest level (i.e., measurement of core affects via psychophysiology, recognition of the emotions expressed, and reflection of what kind of experience the whole process induces in the perceiver), it is fundamentally not a symmetrical and reversible process. One cannot reduce the experience of longing (a complex, aesthetic emotion) into recognition of combination of basic emotions nor predict the exact core affects related to such emotional experience. At best, one level may *modulate* the processes taking place in the lower levels (as depicted with the downward arrows in **Figure 2**). The extent of such top–down influence has not received sufficient attention to date, although top–down information such as extramusical information has been demonstrated to impact music-induced emotions (Vuoskoski and Eerola, 2015). However, such top–down effects on perception are well known in perceptual literature (Rahman and Sommer, 2008) and provide evidence against strictly modular framework. Despite this shortcoming, the hybrid model does organize the range of processes in a functional manner.

## Emotions Modulated by Aesthetic Experience

In what preceded we have emphasized the bottom–up approach to musically induced emotions, taking as a starting point that affective experience may reflect an evolutionary primitive form of consciousness above which more complex layers of consciousness can emerge (Panksepp, 2005). Many higher neural systems are in fact involved in the various distinct aspects of experiencing and recognizing musical emotions, but a great deal of the emotional power may be generated by lower subcortical regions where basic affective states are organized (Panksepp, 1998; Damasio, 1999; Panksepp and Bernatzky, 2002). This lower level processing, however, can be modified to some extent by other variables such as repeated encounters with the stimulus—going from mere exposure, over habituation and sensitization—, co-occurrence with other stimuli (classical and evaluative conditioning) and varying internal states such as, e.g., motivation (Moors, 2007, p. 1241).

A real aesthetic experience of music, moreover, can be defined as an experience “in which the individual immerses herself in the music, dedicating her attention to perceptual, cognitive, and affective interpretation based on the formal properties of the perceptual experience” (Brattico and Pearce, 2013, p. 49). This means that several mechanisms may be used for the processing, elicitation, and experience of emotions (Storbeck and Clore, 2007).

Musical sense-making, in this view, has to be broadened from a mere cognitive to a more encompassing approach that includes affective semantics and embodied cognition. What really counts in this regard, is the difficult relationship between *emotion* and *cognition* (Panksepp, 2009–2010). Cognition, regarded in a narrow account, is contrasted mainly with emotion and cognitive output is defined as information that is not related to emotion. It is coined “cold” as contrasted with “hot” affective information processing (Eder et al., 2007). Recent neuroanatomic studies, however, seem to increasingly challenge the idea of specialized brain structures for cognition versus emotion (Storbeck and Clore, 2007), and there is also no easy separation between cognitive and emotional components insofar as the functions of these areas are concerned (Ishizu and Zeki, 2014). Some popular ideas about cognition and emotion such as *affective independence*, *affective primacy* and *affective automaticity* have been questioned accordingly (Storbeck and Clore, 2007, pp. 1225–1226): the affective independence hypothesis states that emotion is processed independently of cognition via a subcortical low route; affective primacy claims precedence of affective and evaluative processing over semantic processing, and affective automaticity states that affective processes are triggered automatically by affectively potent stimuli commandeering attention. A more recent view, however, is the suggestion that affect modifies and regulates cognitive processing rather than being processed independently. Affect, in this view, probably does not proceed independently of cognition, nor does it precede cognition in time. (Storbeck and Clore, 2007, pp. 1225–1226).

As such, there is some kind of overlap between music-evoked complex and/or “aesthetic emotions” and so-called “everyday emotions” (Koelsch, 2014). Examples of the latter are anger, disgust, fear, enjoyment, sadness, and surprise (see Matsumoto and Ekman, 2009). They are mainly reducible to the basic emotions—also called “primary,” “discrete” or “fundamental” emotions—which have been elaborated in several taxonomies. Examples of the former are wonder, nostalgia, transcendence (see Zentner et al., 2008; Trost et al., 2012; Taruffi and Koelsch, 2014). They are typically elicited when people engage with artworks (including music) and objects or scenes in nature (Robinson, 2009; see Sander, 2013 for an overview) and can be related to “epistemic emotions” such as interest, confusion, surprise or awe (de Sousa, 2008) though the latter have not yet been the focus of much research in affective neuroscience. As explained in the hybrid model (Eerola, 2017), however, they tend to be rare, less stable and more reliant on the various other factors related to meaning-generation in music (Vuoskoski and Eerola, 2012). Related topics, such as novelty processing, have been investigated extensively—with a key role for the function of the amygdala—as well as the role emotions, which are not directed at knowing, can have for epistemic consequences. Fear, for instance, can lead to an increase in vigilance and attention with better knowledge of the situation in order to evaluate the possibilities for escape (Sander, 2013).

The everyday/aesthetic dichotomy, further, is related also to the distinction between *utilitarian* and *aesthetic* emotions (Scherer and Zentner, 2008). The latter occur in situations that do not trigger self-interest or goal-directed action and reflect a multiplicative function of structural features of the music, listener features, performer features and contextual features leading to distinct kinds of emotion such as wonder, transcendence, entrainment, tension and awe (Zentner et al., 2008). It is possible, however, to combine aesthetic and non-aesthetic emotions when asked to describe retrospectively felt and expressed musical emotions. As such, nine factors have been described—commonly known as the *Geneva Emotional Music Scale* or GEMS (see Zentner et al., 2008), namely wonder, transcendence, tenderness, nostalgia, peacefulness, power, joy, tension and sadness. Awe, nostalgia, and enjoyment, among the aesthetic emotions, have attracted the most detailed research with aesthetic awe being crucial in distinguishing a peak aesthetic experience of music from everyday casual listening (Gabrielsson, 2010; Brattico and Pearce, 2013, p. 51), although studies that induce a range of emotions in laboratory conditions may fail to arouse the special emotions such as awe, wonder and transcendence (Vuoskoski and Eerola, 2011).

## Conclusion and Perspectives: Nature Meets Nurture

In this paper, we explored the evolutionary groundings of music-induced emotions. Starting from a definition of emotions as adaptive processes we tried to show that music-induced emotions reflect ancient brain functions. The inductive power of such functions, however, can be expanded or even overruled to some extent by the evolutionary younger regions of the brain. The issue whether an emotional modulation of sensory input is “top–down” and dependent upon input from “higher” areas of the brain or whether it is “bottom–up,” or both, is up to now an unresolved question (Ishizu and Zeki, 2014). Affect and cognition, in fact, have long been treated as independent domains, but current evidence seems to suggest that both are in fact highly interdependent (Storbeck and Clore, 2007). Although we may never know with certainty “the evolutionary and cultural transitions that led from our acoustic-emotional sensibilities to an appreciation of music” it may be suspected that the role of subcortical systems in the way we are affected by music has been greatly underestimated (Panksepp and Bernatzky, 2002, p. 151). Music establishes affective resonances within the brain, and it is within an understanding of the ingrained emotional processes of the mammalian brain that the essential answers to these questions will be found, which could imply that affective sounds are related to primitive reactions with adaptive power and that somehow music capitalizes on these reactive mechanisms. In this view, early affective processing—as relevant in early infancy and prehistory—, should reflect the way the emotions make our bodies feel, which in turn reflects on the emotions expressed and decoded.

Music-induced emotions, moreover, have recently received considerable impetus from neurobiological and psychobiological research. The full mechanisms behind the proposed induction mechanisms, however, are not yet totally clear. Emotional processing holds a hybrid position: it is the place where nature meets nurture with emotive meaning relying both on pre-programmed reactivity that is based on wired-in circuitry for perceptual information pickup (*nature*) and on culturally established mechanisms for information processing and sense-making (*nurture*). It makes sense, therefore, to look for mechanisms that underlie the inductive power of the music and to relate them with evolutionary claims and a possible adaptive function of music. Especially important here is the distinction between the acoustic and the vehicle mode of listening and the related distinction between the on-line and off-line mode of listening. Much more research, however, is needed in order to investigate the relationship between music-specific or aesthetic emotions and everyday or utilitarian emotions (Scherer and Zentner, 2008; Reybrouck and Brattico, 2015). The latter are triggered by the need to adapt to specific situations that are of central significance to the individual’s interests and well-being; the former are triggered in situations that usually have no obvious material effect on the individual’s well-being. Rather than relying on categorical models of emotion by blurring the boundaries between aesthetic and utilitarian emotions we should take care to reflect also the nuanced range of emotive states, that music can induce. As such, there should be a dynamic tension between the “nature” and the “nurture” side of music processing, stressing the role of the musical experience proper. Music, in fact, is a sounding and temporal phenomenon which has inductive power. The latter involves ongoing epistemic interactions with the sounds, which rely on low-level sensory processing as well as on principles of cognitive mediation. The former, obviously, refer to the nature side, the latter to the nurture side of music processing. Cognitive processing, however, should take into account also the full richness of the sensory experience. What we argue for, therefore, is the reliance on the nature side again, which ends up, finally, in what may be called a “*nature-nurture-nature cycle*” of musical sense-making, starting with low-level processing, over cognitive mediation and revaluing the sensory experience as well (Reybrouck, 2008).

## Author Contributions

The first draft of this paper was written by MR. The final elaboration was written jointly by MR and TE.

## Conflict of Interest Statement

The authors declare that the research was conducted in the absence of any commercial or financial relationships that could be construed as a potential conflict of interest.
